# Predictors of 12-Month Recurrence of Hemoptysis after Bronchial Artery Embolization

**DOI:** 10.34172/aim.33457

**Published:** 2025-02-01

**Authors:** Sareh Sadidi, Farzin Roozafzai, Sirous Nekooei, Lida Jarahi, Farzaneh Khoroushi

**Affiliations:** ^1^Department of Radiology, Mashhad University of Medical Sciences, Mashhad, Iran; ^2^Digestive Diseases Research Institute, Tehran University of Medical Sciences, Tehran, Iran; ^3^Department of Community Medicine, Mashhad University of Medical Sciences, Mashhad, Iran

**Keywords:** Bronchial artery embolization, Hemoptysis, Recurrence, Risk factor

## Abstract

**Background:**

Despite the high success rate of bronchial artery embolization (BAE), hemoptysis probably recurs. This study investigated risk factors of 12-month hemoptysis recurrence after BAE in an Iranian population.

**Methods:**

In this prospective cohort, we followed up 101 patients for 12 months after BAE. Outcome of interest was recurrence of hemoptysis. Target arteries were super-selectively catheterized and embolized with non-spherical polyvinyl alcohol particles (150–700 µm). Success of BAE was confirmed using post-BAE angiography. Independent t-test, and chi-square and Fisher’s exact test were used to compare variables between "recurrence" and "non-recurrence" groups. We investigated predictors of recurrent hemoptysis through univariate and multivariate logistic regression modeling. We analyzed receiver operating characteristic curve to find the optimal cutoff point for continuous risk factors. Recurrence-free rates stratified by risk factors were plotted against time using the Kaplan-Meier method.

**Results:**

BAE was immediately successful in all patients. During the 12-month follow-up, hemoptysis recurred in 13.9% (95% CI: 8.2–21.6) of participants. Mean (±standard deviation) recurrence-free time was 6.9 (±3.3) months. Lung destruction (OR=5.40 [95% CI: 1.41–20.58], *P* value=0.013) and arterial diameter≥2 mm (12.51 [1.51–103.59], *P* value=0.019) were independent predictors of 12-month hemoptysis recurrence.

**Conclusion:**

Patients with destroyed lungs and embolized arteries wider than 2.0 mm are at higher risk of hemoptysis recurrence in the first year after BAE.

## Introduction

 Hemoptysis, expectoration of blood from the respiratory system, is a common and potentially life-threatening symptom.^1-3^ The etiology of hemoptysis varies regionally: the most common causes are tuberculosis (TB) and bronchiectasis in Eastern countries, and lung cancer in Western countries.^3-6^ Bronchiectasis is a chronic respiratory disease with an increasing worldwide prevalence.^7^ Unfortunately, no data on the epidemiology of bronchiectasis is available from regions such as the Middle East.^8^ Neglected or incompletely treated pulmonary infections, such as TB, are the most common etiology of bronchiectasis in low-and-middle-income countries.^9^ TB is intermediately prevalent in Iran, particularly in eastern provinces neighboring Afghanistan and Pakistan where TB is highly prevalent and incident.^10-12^ Social determinants of health such as low socioeconomic status, cultural stigma, illiteracy, or limited access to diagnostic and therapeutic facilities, might make the population of those eastern provinces leave the hemoptysis untreated.^13-15^ The mortality rate of untreated or conservatively managed massive hemoptysis ( > 300 mL/d) is greater than 50%.^2-5,16,17^

 Bronchial arteries are the most common anatomic source of hemoptysis.^1,2,18^ Bronchial artery embolization (BAE) is recommended as the first-line treatment of hemoptysis, particularly in emergency settings, and high-risk or inoperable patients.^1^ Advances in BAE techniques, such as super-selective coaxial microcatheterization, particulate embolization, and pre- and post-embolization angiography, have led to more effective control of hemoptysis.^16,19^ Despite high immediate clinical success rate, defined as complete cessation of hemoptysis after BAE, recurrence rate remains high and up to 57% of patients might experience recurrent hemoptysis after BAE.^4^ Incomplete BAE, bleeding from non-bronchial arteries, parenchymal lung destruction, size of embolized arteries, recanalization, and neovascularization, have been previously reported as predictors of hemoptysis recurrence.^1,4,16,20-24^

 The high recurrence rate necessitates understanding of risk factors, tailoring treatment plans, and optimizing hemoptysis management strategies. We aimed to investigate risk factors predicting the recurrence of hemoptysis after BAE in a population of north-eastern Iranians whom the well-known etiologies of hemoptysis, such as TB and bronchiectasis, might prevalently affect.

## Materials and Methods

###  Patients and Design

 In this prospective observation, we enrolled all consecutive patients who underwent BAE for hemoptysis in two tertiary-care university hospitals in Mashhad, Iran, between 2017 and 2022. We consequently followed up the participants for 12 months looking for recurrence of hemoptysis after BAE. Subjects would be excluded if they: disagreed to participate; were younger than 18 years; had missing data or imaging; withdrew; or were lost to follow-up.

 Assuming a hemoptysis recurrence rate (p) of 10%,^1^ a margin of error (δ) of 6%, type I error (α) of 5%, and an expected dropout rate of 30%, an approximate size (N) of 127 subjects for the sample population was calculated using the following formula:


$$N=Z_{1-\alpha /2}^{2}\left[p\left(1-p\right)\right]/\delta^{2};\;\alpha =0.05,\;p=0.1,\;\delta =0.06$$

###  Baseline Data

 In the enrollment phase (2017–2022), we interviewed the participants, reviewed medical documents and radiologic images, and collected baseline data regarding demographics (age, sex, tobacco smoking), etiologic disease (TB, cancer, chronic obstructive pulmonary disease, and other etiologies), paraclinical assessments (prothrombin time and partial thromboplastin time), and radiologic findings. Contrast-enhanced thoracic computed tomography (CT) and CT-angiography findings included location, number, and maximum diameter of target arteries, and presence of bronchiectasis, emphysema, honeycombing, consolidation, mass, cavity, collapse, fibrosis, and destructive changes in lungs. Lung destruction was defined as diffuse parenchymal destruction or volume loss in at least one lobe.^21^

###  CT-angiography

 Before BAE, CT-angiography was performed using NeuViz 16-slice CT scanner (Neusoft Corp., Shenyang, China) with 2.0 cc/kg of Visipaque (Iodixanol 320 mgI/mL; GE Healthcare, Chicago, IL, USA) injected into the antecubital vein at a speed of 4.0 mL/s. Then, the data were transferred to a post-processing workstation, reconstructed at 2.0 mm section thickness, and examined by two interventional radiologists. The CT scanner used in this study is calibrated annually by Parsian Radiation Dosimetry Services Company, Tehran, Iran. In the last quality control tests (2023, reference number: LQF-510-08), the rate of leakage/scatter of dye ( < 1 mGy/h) and the precision of CT numbers (error < 5 Hounsfield unit) were acceptable.

###  Bronchial Artery Embolization 

 Two experienced interventional radiologists performed all procedures via a transfemoral approach under local anesthesia using 4-F or 5-F guide catheters (Merit Medical, South Jordan, UT, USA). To confirm the location of bleeding, a flush descending thoracic aortogram, opacifying both bronchial and non-bronchial systemic arteries, was acquired at the beginning of BAE. This non-selective arteriography was followed by super-selective catheterization of target arteries introducing and carefully advancing a 2.9-F coaxial microcatheter (Merit Medical, South Jordan, UT, USA), to avoid non-target embolization of side branches, such as anterior spinal artery. Non-spherical polyvinyl alcohol (PVA) particles (Merit Medical, South Jordan, UT, USA), 150–700 microns in size, were injected until the proximal portion of the artery was occluded on angiography. All abnormal vessels supplying the area of interest were embolized if technically possible. No patient underwent coil embolization in our cohort. A successful BAE would significantly reduce and cease hemoptysis, and would be confirmed through a post-BAE angiography showing no blushing or distal vascular signs (technical success), with no recurrence of hemoptysis within the same admission (clinical success).^3,25^

###  Outcome of Interest

 Recurrence of hemoptysis was defined as clinically significant hemoptysis ( ≥ 30 mL/d) occurring after clinical success and requiring clinical management.^21^

###  Follow-up Data

 After 12 months, we actively followed-up each participant through phone calls and inquired about recurrent hemoptysis or cause-specific death, and their pertinent dates. In case of death, we would ask the participant’s family members, care-givers, or disease registries to report the cause of death and recurrence of hemoptysis, and provide the available medical documents (e.g. death certificate, or autopsy report).

###  Endpoints

 Endpoints of the study were death, withdrawal, and loss to follow-up. Participants who did not answer three consecutive calls in a week would be considered lost.

###  Statistical Analysis

 According to the follow-up data, participants were divided into recurrence and non-recurrence groups. We used descriptive statistics to report categorical variables (frequency distributions) and continuous variables (mean and standard deviation [SD]). We respectively used independent t-test, and chi-square and Fisher’s exact test, to compare continuous and categorical variables between recurrence and non-recurrence groups. We investigated variables predicting recurrent hemoptysis through univariate and multivariate Cox logistic regression modeling. Significant univariate predictors were inserted in the multivariate models with the backward selection method. Models were summarized in terms of odds ratio (OR) with 95% confidence interval (CI). We also plotted receiver operating characteristic (ROC) curve and used the Youden index to determine the optimal cutoff point for continuous risk factors. Sensitivity, specificity, positive predictive value (PPV), negative predictive value (NPV), accuracy, positive likelihood ratio (LR + ), negative likelihood ratio (LR-), and diagnostic odds ratio (DOR) were calculated for the selected cutoff points. We used the Kaplan-Meier method to plot recurrence-free probabilities against time in different categories of risk factors, and compared the categories using the Cox-Mantel log-rank test. We did not investigate potential confounding factors, such as socioeconomic status, comorbidities, or medications. The data were analyzed using IBM SPSS v.25 (IBM corp., Chicago, IL, USA). Level of significance was 0.05.

## Results

**Figure 1:**
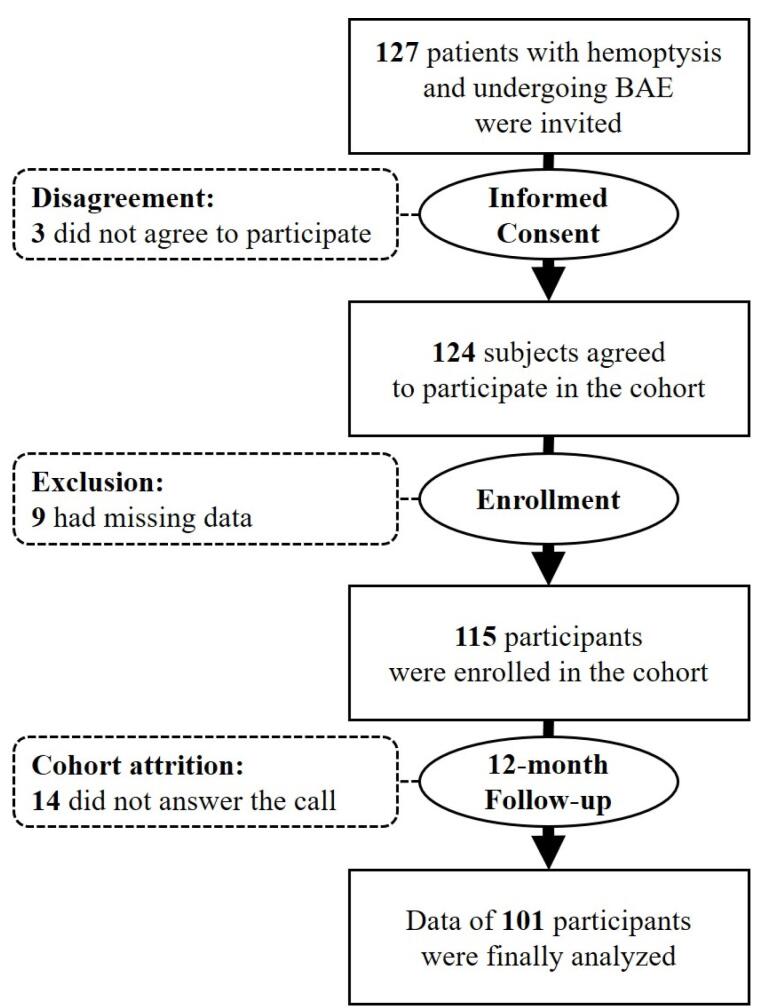
Schematic Flowchart of the Study. BAE: bronchial artery embolization

 Of 127 recruited patients (76 men and 51 women), we excluded three unwilling subjects and nine with missing data. We collected the baseline data of 115 participants in the enrollment and followed up the participants for 12 months. Attrition of the cohort included 14 participants who did not answer the follow-up phone calls. We finally analyzed the data of 101 participants comprising 64 men (63.4%) and 37 women (36.6%). Figure 1 shows the study flowchart.

 Hemoptysis was successfully controlled immediately after BAE in all patients. During the 12-month follow-up, hemoptysis recurred in 14 cases (recurrence group; 13.9% [95% CI: 8.2–21.6]) with a mean ( ± SD) recurrence-free duration of 6.9 ( ± 3.3) months. The other 87 participants were recurrence-free after 12 months (non-recurrence group; 86.1% [95% CI: 78.4–91.8]). Cumulative hemoptysis recurrence-free rates at the first, third, sixth, and ninth months after BAE were 1.00, 0.97, 0.93, and 0.91, respectively.

 There was no significant difference in sex distribution between the recurrence and non-recurrence groups (*P* value = 1.000). The mean ( ± SD) age was 61.0 ( ± 15.9) years, showing no significant difference between the recurrence and non-recurrence groups (Table 1).

**Table 1:** Comparison of Baseline Characteristics in Recurrence and Non-recurrence Groups of Patients with Hemoptysis Treated by Bronchial Artery Embolization

**Characteristics**		**Total (n=101)**	**Recurrent Hemoptysis**		**Difference (95% CI)**^c^	***P*** Value^*^
			**Yes (n=14)**	**No (n=87)**		
		**Mean±Standard Deviation**				
Age (y)		61.0 ± 15.9	65.0 ± 16.2	60.4 ± 15.9	4.5 (-4.5–13.6)	0.327
Diameter of target artery (mm)		2.0 ± 0.5	2.2 ± 0.3	1.9 ± 0.5	0.31 (0.01–0.60)	0.040
PT (second)		13.6 ± 2.4	13.6 ± 1.8	13.7 ± 2.5	-0.1 (-1.5–1.2)	0.870
PTT (second)		29.9 ± 16.6	25.3 ± 4.0	30.7 ± 17.8	-5.3 (-14.9–4.13)	0.264
			**Count (Percent)**^a^			***P*** Value^**^
Age ≥ 65 years		47 (46.5)	8 (57.1)	39 (44.8)	12.3 % (-15.6–40.2)	0.406
Diameter of target artery ≥ 2 mm		58 (57.4)	13 (92.9)	45 (51.7)	41.2 % (24.0–58.2)	0.003
Male sex		64 (63.4)	9 (64.3)	55 (63.2)	1.1 % (-25.9–28.1)	1.000
Tobacco smoking		27 (26.7)	6 (42.9)	21 (24.1)	18.8 % (-8.6–46.2)	0.192
TB		31 (30.7)	7 (50.0)	24 (27.6)	22.4 % (-5.4–50.2)	0.120
Cancer ^b^		11 (10.9)	3 (21.4)	8 (9.2)	12.2 % (-10.1–34.5)	0.178
Lung cancer		6 (5.9)	2 (14.3)	4 (4.6)	9.7 % (-9.1–28.5)	0.193
COPD		7 (6.9)	0 (0.0)	7 (8.0)	-8.0 % (-13.7–-2.3)	0.589
Emphysema		26 (25.7)	2 (14.3)	24 (27.6)	13.3 % (-33.9–7.3)	0.510
Bronchiectasis		58 (57.4)	11 (78.6)	47 (54.0)	24.6 % (0.7–48.5)	0.144
Honeycombing		2 (1.9)	1 (7.1)	1 (1.1)	6.0 % (-7.6–19.6)	0.259
Consolidation		47 (46.5)	8 (57.1)	39 (44.8)	12.3 % (-15.6–40.2)	0.406
Collapse		15 (14.9)	4 (28.6)	11 (12.6)	16.0 % (-8.6–40.6)	0.216
Fibrotic changes		44 (43.6)	10 (71.4)	34 (39.1)	32.3 % (6.5–58.1)	0.039
Destructive changes		17 (16.8)	6 (42.9)	11 (12.6)	30.3 % (3.3–57.0)	0.012
Cavity		33 (32.7)	7 (50.0)	26 (29.9)	20.1 % (-7.8–48.0)	0.217
Mass		13 (12.9)	3 (21.4)	10 (11.5)	9.9 % (-12.6–32.4)	0.383
Size of PVA particle	150–300 µm	17 (16.8)	3 (21.4)	14 (16.1)	5.3 % (-17.5–28.1)	0.849
	300–500 µm	60 (59.4)	8 (57.1)	52 (59.8)	-2.7 % (-30.6–25.2)	
	500–700 µm	24 (23.8)	3 (21.4)	21 (24.1)	-2.7 % (-25.9–20.5)	
Embolized artery	Bronchial	38 (37.6)	3 (21.4)	35 (40.2)	-18.8 % (-42.6–5.0)	0.155
	Non-bronchial	9 (8.9)	0 (0.0)	9 (10.3)	-10.3 % (-16.6–-3.9)	
	Both	54 (53.5)	11 (78.6)	43 (49.4)	29.2 % (5.2–53.1)	
Number of embolized arteries	1 artery	28 (27.7)	1 (7.1)	27 (31.0)	-23.9 % (-40.5–-7.3)	0.074
	2 arteries	44 (43.6)	6 (42.9)	38 (43.7)	-0.8 % (-28.7–27.1)	
	≥ 3 arteries	29 (28.7)	7 (50.0)	22 (25.3)	24.7 % (-3.0–52.4)	

CI: confidence interval, COPD: chronic obstructive pulmonary disease, n: count, PT: prothrombin time, PTT: partial thromboplastin time, PVA: polyvinyl alcohol, TB: tuberculosis. ^*^Independent samples t-test; level of significance < 0.05 ^**^Fisher’s exact test; level of significance < 0.05 ^a^Numbers represent frequency distributions in the column. ^b^Including lung cancer, metastatic cancer of other origins, and lymphoma. ^c^Calculated by subtracting values in “No” column from values in “Yes” column.

 As represented in Table 1, significant differences were found in distribution of destructive and fibrotic changes, and maximum diameter of embolized artery between the recurrence and non-recurrence groups. Lung destruction (42.9% [95% CI: 20.3–68.1]) and fibrotic changes (71.4% [45.5–89.5]) were more prevalent in the recurrence group than in the non-recurrence group (12.6% [6.9–20.8] and 39.1% [29.3–49.6], respectively); and, the diameter of embolized artery was 0.31 mm (95% CI: 0.01–0.60) wider in the recurrence group.

**Figure 2:**
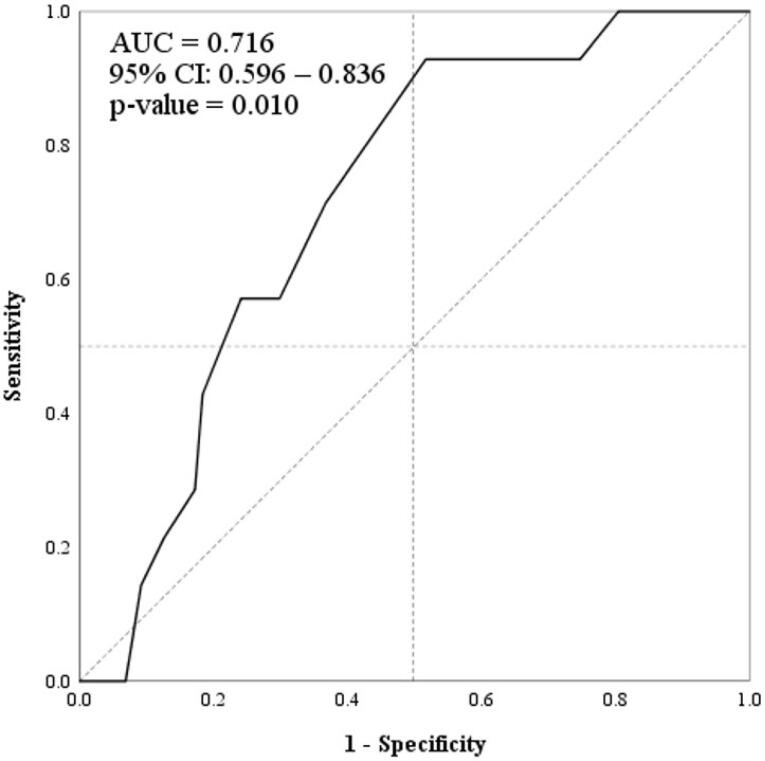
Receiver Operating Characteristic Curve of “Maximum Diameter of Embolized Artery” (Solid Line) for Discriminating “12-month Hemoptysis Recurrence”. AUC: area under the curve, CI: confidence interval

 Based on the ROC curve (Figure 2), we selected 2.0 mm (Youden index = 0.412) as the cutoff for maximum arterial diameter for discriminating the recurrence. The sensitivity, specificity, PPV, NPV, accuracy, LR + , LR-, and DOR for the selected cutoff point were 92.8%, 48.2% 22.4%, 97.6%, 54.4%, 1.79, 0.14, and 12.13, respectively. Further details are represented in Table 2.

**Table 2:** Sensitivity, Specificity, and Predictive Abilities of Four Different Cut-offs for Arterial Diameter for Discriminating “12-month Hemoptysis Recurrence” in 101 Patients Treated by Bronchial Artery Embolization

		**Cut-off for Arterial Diameter**							
		**≥1.5 mm** **(Most Sensitive)**		**≥2.0 mm** **(Selected)**		**≥3.0 mm** **(Predefined)** ^a^		**≥4.0 mm** **(Most Specific)**	
		**+**	**-**	**+**	**-**	**+**	**-**	**+**	**-**
Recurrent hemoptysis	+	14	0	13	1	0	14	0	14
	-	70	17	45	42	5	82	0	87
Sensitivity (%)		100.0		92.8		0.0		0.0	
Specificity (%)		19.5		48.2		94.2		100.0	
PPV (%)		16.6		22.4		0.0		–^*^	
NPV (%)		100.0		97.6		85.4		86.1	
LR +		1.24		1.79		0.0		–^*^	
LR-		0.0		0.14		1.06		1.00	
DOR		–^*^		12.13		0.0		–^*^	

DOR: diagnostic odds ratio, LR + : positive likelihood ratio, LR-: negative likelihood ratio, NPV: negative predictive value, PPV: positive predictive value. ^*^Unable to compute (division by zero). ^a^The diameter regarded as abnormal in the literature.^19^

**Figure 3:**
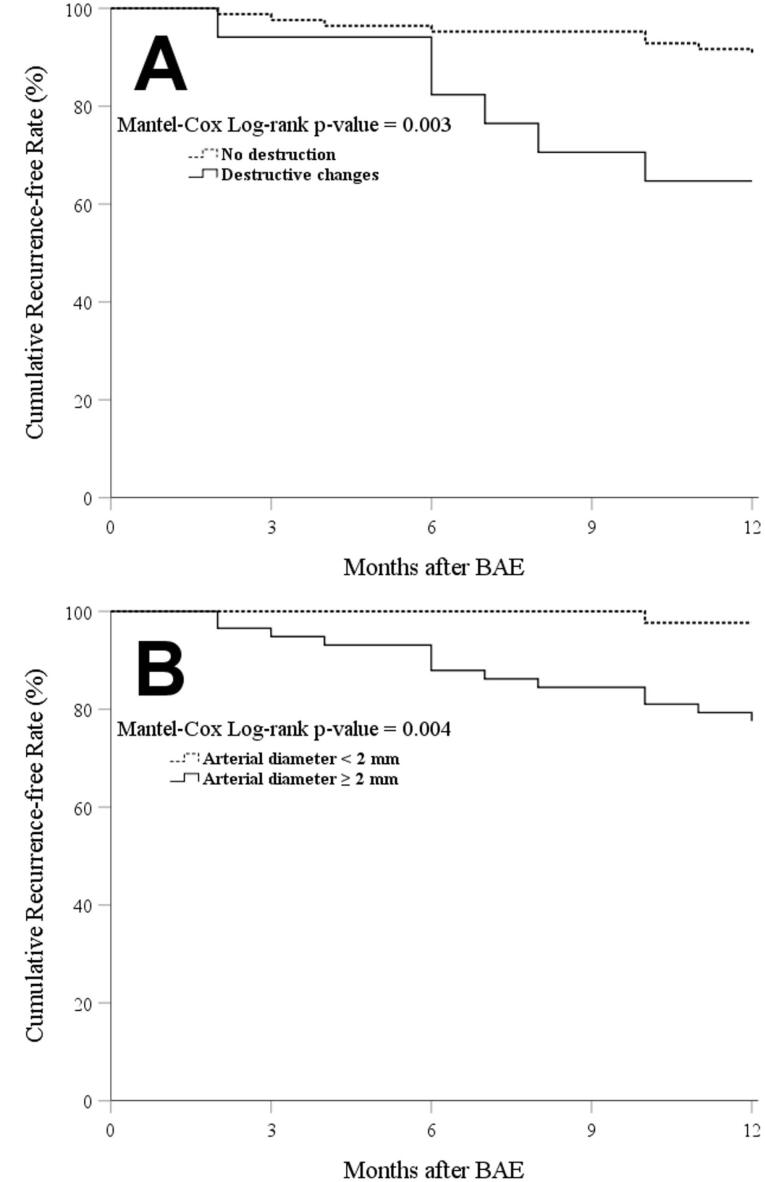
Kaplan–Meier Curves for Cumulative Hemoptysis Recurrence-free Rates Based on: (**A**) Presence of Lung Destruction; and (**B**) Maximum Diameter of Embolized Artery. BAE: bronchial artery embolization

 In univariate logistic regression models, fibrosis, destructive changes, and maximum arterial diameter significantly predicted the 12-month recurrence of hemoptysis. Yet, multivariate models showed that only destructive changes (OR = 5.40 [95% CI: 1.41–20.58]) and arterial diameter ≥ 2 mm (12.51 [1.51–103.59]) were independently associated with the 12-month recurrence (Table 3). We also found destructive changes (9.47 [2.04–43.91]) and arterial diameter ( + 1 mm increments, 4.42 [1.03–19.00]) as independent predictors of 9-month recurrence. Figure 3 demonstrates the Kaplan-Meier curves for recurrence-free time in the participants based on destructive changes and arterial diameter.

**Table 3:** Significant Univariate and Multivariate Predictors of 12-months Hemoptysis Recurrence after Bronchial Artery Embolization

**Predictor**	**Univariate Model**		**Multivariate Model **^a^	
	**OR (95% CI)**	***P*** ** Value**	**OR (95% CI)**	***P*** ** Value**
Diameter of artery ≥ 2 mm	12.13 (1.52–96.82)	0.019	12.51 (1.51–103.59)	0.019
Destructive changes	5.18 (1.51–17.78)	0.009	5.40 (1.41–20.58)	0.013
Fibrotic changes	3.89 (1.13–13.42)	0.031		

CI: confidence interval, OR: odds ratio. ^a^Multivariate Cox logistic regression modeling with backward selection method.

## Discussion

 Hemoptysis has different etiologies in different regions. Consistent with previous observations in Iran and other Eastern countries, TB (30.7%) and bronchiectasis (57.4%) were the most prevalent etiologies of hemoptysis in our cohort.^4,21,26,27^ We studied a population in northeastern Iran, where various socioeconomic factors might constrain the affected people to neglect their hemoptysis or to delay the clinical visit, which consequently might lead to more severe morbidity, and higher rates of treatment failure or hemoptysis-related mortality.^13-17^

 In the current study, bleeding was immediately controlled in all patients. According to the literature, the overall immediate clinical success rate of BAE is 70%–100%.^1,19^ With technologic advances and in the hands of an expert and skilled interventionist, high technical success rates can be achieved.^19,28^ The hemoptysis control rate was approximately 100% after one month and 86% after one year in our cohort.

 Recurrence of hemoptysis after a successful BAE is not uncommon, ranging from 10% to 57%.^1,19^ Despite high immediate clinical success rate in our cohort, hemoptysis recurred in 13.9% (95% CI: 8.2–21.6) of participants during 12 months of follow-up. Previous studies in Iran reported 12-month recurrence rates of 15.5%^26^ and 36.7%,^29^ which is higher than our observation. The rate might be different due to differences in the sample population, research method, BAE technique, and embolizing material.^22^ The former recurrence rate was observed after BAE without microcatheterization, and the latter was observed in patients with pulmonary TB.

 So far, diverse predictors of hemoptysis recurrence are reported. The etiology of hemoptysis was not associated with the recurrence rate in our study. As we observed, destructive changes in the lung and the diameter of the culprit artery were independently associated with the recurrent hemoptysis. Previous studies concluded that hemoptysis would recur due to incomplete embolization, non-bronchial systemic artery involvement, recanalization of embolized arteries, and neovascularization due to inflammation and underlying disease progression.^1,4,16,20,21^ Incomplete embolization and non-bronchial systemic artery involvement were risk factors for early recurrence ( < 1 month), while recanalization and neovascularization were associated with late recurrence ( > 1 month).^20^

 The diameter of the embolized artery was independently associated with the recurrence of hemoptysis in our cohort. As shown previously, relatively large embolized arteries, compared with smaller arteries, were more susceptible to recanalization after BAE.^23^ Generally, a bronchial artery diameter larger than 3 mm is considered abnormal.^19^ We detected 2 mm as the optimal cutoff for arterial diameter for discriminating recurrent hemoptysis, and found that a diameter larger than 2 mm was the strongest predictor of 12-month recurrence.

 Although TB, bronchiectasis, or cancer, as the etiologies, were not significantly associated with recurrence in our cohort, we found that the hemoptysis would probably recur if the lung parenchyma was destructed. This observation was in line with the studies by Kim et al^22^ and Wang et al^24^ which reported lung destruction as a risk factor of recurrent hemoptysis after BAE in patients with pulmonary TB. According to Wang et al, post-TB bronchiectasis patients with destructive changes were at quadruple higher risk of recurrent hemoptysis than patients without destroyed lung.^24^ Lu et al also found lung destruction as a predictor of early recurrence of hemoptysis.^20^ On the contrary, TB-destroyed lung was not associated with recurrent hemoptysis ina study byHwang et al^30^ This discrepancy might emanate from differences in BAE technique, TB prevalence, study timeframe, and demographics of studied populations.^22^ TB-destroyed lung was found to be a poor prognostic factor of hemoptysis that is associated with decreased lung function and increased mortality.^22,24^

 Apart from methodological limits, such as information bias, a draw-back of the present study is lack of data pertaining to the volume and severity of hemoptysis, and potential confounding factors, such as socioeconomic status, comorbidities (e.g. diabetes mellitus, and chronic liver diseases), and medical treatment for etiologic diseases (before and after BAE). We did not assess inflammatory markers, since they are highly variable in long-term.^21^ We followed recommended standards for BAE (device calibration, use of PVA, super-selective embolization, and post-BAE angiography) to reduce technical bias.^1^ To minimize the information bias, two radiologists independently assessed the images, and the quantitative data (e.g. arterial diameter) were averaged. There were no disparities in assessment of qualitative features (e.g. presence of destructive changes) by the two radiologists (not reported). The radiologists, unaware of the recurrence status, were not blinded to the etiologic disease, or technical and clinical outcomes of BAE.

## Conclusion

 During the first year after BAE, hemoptysis recurred in 13.9% of the participants in this cohort. According to our observation, patients should be followed up at least one year after BAE, particularly cases with destroyed lungs and embolized arteries wider than 2.0 mm. Further studies with larger sample sizes might corroborate our observation, and are needed to investigate factors affecting long-term recurrence of hemoptysis.
